# Editorial: Bone-organ axis: an expanding universe

**DOI:** 10.3389/fendo.2025.1574370

**Published:** 2025-06-05

**Authors:** Peilin Zhang, Yicheng Wang, Yijia Guo, Xiaonan Liu

**Affiliations:** Department of Orthopaedics, Shanghai Sixth People’s Hospital Affiliated to Shanghai Jiao Tong University School of Medicine, Shanghai, China

**Keywords:** bone metabolism, degenerative diseases, musculoskeletal disorder, organ crosstalk, endocrine dysfunction

The musculoskeletal system, long regarded as a biomechanical scaffold, is increasingly recognized as a dynamic player in systemic physiology, profoundly interconnected with other organ systems (**Figure 1**). From a physiological perspective, bone functions as an endocrine organ that coordinates bodily functions, thereby enhancing the perception of the surrounding environment and improving athletic performance (1). Pathologically, degenerative musculoskeletal diseases such as osteoporosis, osteoarthritis (OA) and intervertebral disc degeneration (IVDD) are not merely localized bone/cartilage disorders but manifestations of broader systemic imbalances such as metabolic syndrome (2), sarcopenia (3, 4), senescence (5), and systemic inflammation. The crosstalk between bone and other organs—mediated by a complex interplay of biochemical signals, metabolic pathways, and endocrine functions—offers new perspectives on the pathogenesis of these diseases and potential avenues for therapeutic innovation. This Research Topic of *Frontiers in Endocrinology* explores these intricate relationships, with a particular emphasis on the bone-organ axis.

**Figure 1:**
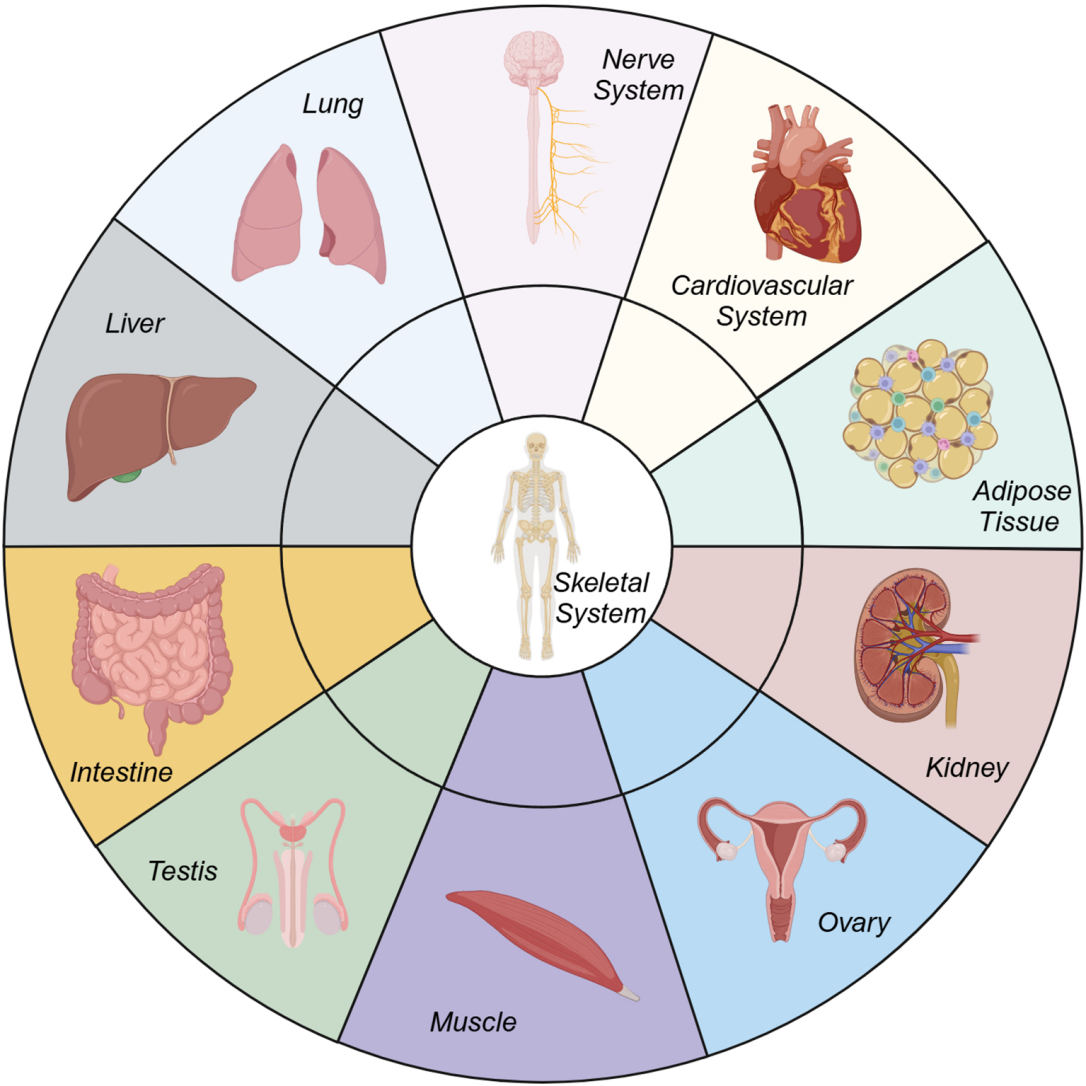
Crosstalk between bone and other organs.

## The bone-endocrine interface: a central regulator

Osteokines such as osteocalcin, fibroblast growth factor 23 (FGF23), and sclerostin serve as critical mediators in the crosstalk between bone and other organs. For instance, osteocalcin, particularly its undercarboxylated form, has been shown in animal studies to regulate glucose metabolism and insulin sensitivity in peripheral tissues, thereby establishing a link between bone health and metabolic disorders like diabetes mellitus (DM) (6, 7). Of note, these effects have yet to be conclusively demonstrated in human. Conversely, metabolic derangements seen in DM, such as chronic hyperglycemia and advanced glycation end products (AGEs), impair bone remodeling, increase fracture risk, and exacerbate degenerative joint diseases (8, 9). In this Research Topic, the link between metabolic dysfunction and bone disorders was revealed in several publications. Azami et al. conducted a systematic review that underscored the reciprocal relationship by identifying musculoskeletal complications as underdiagnosed yet significant comorbidities in DM. Additionally, Zhang et al. demonstrated an association between primary frozen shoulder and dyslipidemia, especially in individuals with underlying conditions such as diabetes and thyroid dysfunction, suggesting a potential metabolic contribution to its pathogenesis. Furthermore, Fu et al. reviewed the mechanisms by which gut microbiota metabolites influence bone health. Collectively, these findings advocate for a comprehensive approach to musculoskeletal health that incorporates metabolic regulation as a fundamental component.

## Bone-muscle crosstalk: a synergistic partnership

The relationship between bone and muscle extends beyond mere mechanical interaction to encompass biochemical and endocrine communication. Myokines, such as irisin, which are released during muscle contraction, play a significant role in influencing bone density and remodeling by promoting osteoblast activity (10). Conversely, signals derived from bone, including transforming growth factor-beta (TGF-β) and sclerostin, modulate muscle mass and function (11). This bidirectional relationship highlights the interdependence of muscle and bone in the maintenance of musculoskeletal integrity. Disruption of this balance contributes to conditions such as sarcopenia and osteoporosis, which frequently coexist and exacerbate the progression of degenerative diseases. In their review of the current literature, Hurly-Novatny et al. summarized the implications of Duchenne muscular dystrophy (DMD) on bone complications. Li et al. investigated the relationship between degeneration of cervical intervertebral disc and paravertebral muscles. Additionally, Jiang et al. explored the potential link between fat infiltration in paraspinal muscles and spinal degeneration.

## The bone-adipose tissue axis: inflammatory mediators in degeneration

Adipose tissue plays a dual role in musculoskeletal health through the secretion of adipokines like leptin, adiponectin, and resistin. While leptin enhances osteoblast activity and bone formation under physiological conditions, its overexpression in obesity contributes to low-grade systemic inflammation, accelerating bone resorption and cartilage degeneration (12–14). In the context of IVDD, He et al. reviewed the role of adipokines in mediating the inflammatory and catabolic cascades that disrupt intervertebral disc homeostasis​. Adiponectin, traditionally regarded as protective, displays context-dependent effects, highlighting the nuanced interplay between adipose tissue and skeletal structures. Targeting this axis through modulation of adipokine signaling holds promise for mitigating the impact of obesity on degenerative musculoskeletal diseases.

## Bone and the central nervous system: the neural-endocrine loop

Emerging research also highlights a neural-endocrine connection between bone and the central nervous system (CNS). Sensory neurons in bone regulate bone remodeling via neuromodulators such as calcitonin gene-related peptide (CGRP) and substance P (15–17). Simultaneously, bone-derived osteocalcin crosses the blood-brain barrier, influencing memory and mood through CNS pathways. In degenerative conditions like OA and IVDD, chronic pain originating from bone and joint pathology can alter neural signaling, leading to a feedback loop of neurogenic inflammation that exacerbates tissue damage (18, 19). Understanding this connection opens the door to integrated treatments addressing both nociceptive and systemic contributors to disease progression.

## Bone and other organs: the expanding horizon

The influence of bone extends beyond muscle, adipose tissue, and the central nervous system to encompass additional physiological systems. Bone marrow functions as a reservoir for immune cells, while inflammatory mediators, such as tumor necrosis factor-alpha (TNF-α), exert a direct impact on bone resorption (20). Chronic systemic inflammation, commonly observed in autoimmune diseases, accelerates degenerative changes in both bone and cartilage. Notably, a Mendelian randomization study conducted by Li et al. established a link between hepatitis B virus (HBV) infection and the development of osteoporosis. Furthermore, bone-derived fibroblast growth factor 23 (FGF23) plays a critical role in regulating phosphate homeostasis and vitamin D metabolism, thereby creating a feedback loop with renal function (21, 22). Impaired renal function disrupts mineral metabolism, which contributes to bone loss and vascular calcification. Vascular calcification, a characteristic feature of atherosclerosis, is associated with dysregulated pathways of bone mineralization, mediated by common factors such as matrix Gla-protein and osteoprotegerin (23). These interconnected pathways highlight the systemic nature of both cardiovascular and skeletal diseases. Additionally, the role of bone in reproductive health has gained increasing attention. Osteocalcin, beyond its metabolic functions, has been implicated in the regulation of male gonadal function. Notably, Oury et al. demonstrated that osteocalcin acts through a pancreas-bone-testis axis to influence testosterone synthesis and fertility in both mice and humans (24). This emerging axis underscores the endocrine capacity of bone in orchestrating systemic physiological processes beyond traditional musculoskeletal functions.

## Implications for precision medicine

The insights presented in this Research Topic emphasize the need for a systemic perspective in understanding and treating degenerative musculoskeletal diseases. Integrating bone health into the broader context of metabolic, endocrine, and inflammatory regulation offers a paradigm shift in degenerative disease management. Thus, the future of research lies in unraveling the molecular mechanisms of bone-organ interactions using advanced technologies like single-cell sequencing and organ-on-a-chip models. Longitudinal studies are needed to establish causal links and identify early biomarkers of systemic dysregulation. Finally, translating these findings into clinical practice requires multidisciplinary collaborations that integrate endocrinology, orthopedics, rheumatology, and metabolic medicine. By recognizing the bone-organ axis as a central player in systemic health, this Research Topic of *Frontiers in Endocrinology* sets the stage for holistic approaches to understanding and managing degenerative musculoskeletal diseases.
